# Persistent major alopecia following adjuvant docetaxel for breast cancer: incidence, characteristics, and prevention with scalp cooling

**DOI:** 10.1007/s10549-018-4855-2

**Published:** 2018-06-19

**Authors:** M. Martín, J. C. de la Torre-Montero, S. López-Tarruella, K. Pinilla, A. Casado, S. Fernandez, Y. Jerez, J. Puente, I. Palomero, R. González del Val, M. del Monte-Millan, T. Massarrah, C. Vila, B. García-Paredes, J. A. García-Sáenz, A. Lluch

**Affiliations:** 1 Medical Oncology Service, Hospital General Universitario Gregorio Marañón, Instituto de Investigación Sanitaria Gregorio Marañón, CIBERONC, GEICAM, Universidad Complutense, Madrid, Spain; 2 Medical Oncology Department, Hospital Clínico San Carlos, Instituto de Investigación Sanitaria del Hospital Clínico San Carlos (IdISSC), CIBERONC, Madrid, Spain; 3 Universidad Pontificia Comillas, Madrid, Spain; 4 Medical Oncology Service, Hospital General Universitario Gregorio Marañón, Instituto de Investigación Sanitaria Gregorio Marañón, CIBERONC, Madrid, Spain; 5 Medical Oncology Department, Hospital Clínico Universitario, CIBERONC, Valencia, Spain; 6 Hospital General Universitario Gregorio Marañón, Medical Oncology Service, Calle Maiquez, no. 9, 28007 Madrid, Spain

**Keywords:** Alopecia, Breast cancer, Docetaxel, Scalp cooling

## Abstract

**Background:**

Persistent alopecia (PA) after docetaxel has been recently described. The aim of our study is to establish the incidence and characteristics of PA following adjuvant docetaxel for breast cancer (BC) and to test the ability of scalp cooling in prevention.

**Patients and methods:**

BC patients receiving adjuvant chemotherapy followed or not by endocrine therapy (and a control group receiving only endocrine therapy) were interviewed in a single institution at 1.5 to 5 years following primary diagnosis searching for PA. A confirmatory prevalence study was later performed in other two institutions. Finally, a prevention study using prophylactic scalp cooling (PSC) with ELASTO-GEL hypothermia caps in patients receiving adjuvant docetaxel was performed.

**Results:**

In the initial prevalence study (492 patients), minor forms of PA (grade 1) were recorded with all chemotherapy regimens and aromatase inhibitors. Patients receiving docetaxel regimens at cumulative dose (CD) ≥ 400 mmg/m^2^ presented a significantly higher prevalence of grades 1 PA (33–52%) and 2 PA (5–12%). Prevalence of grade 2 PA with docetaxel CD ≥ 400 mmg/m^2^ was confirmed in two other institutions. Overall, grade 2 PA was seen in 10.06% (95% CI 7.36–13.61) of 358 patients with docetaxel regimens reaching CD ≥ 400 mmg/m^2^, but not in patients with lower docetaxel CD, other chemotherapy regimens, or endocrine therapy alone. In prevention trial, no grade 2 PA occurred among 116 patients receiving adjuvant docetaxel (≥ 400 mmg/m^2^) and PSC followed-up after a 96 months median time. PSC was well tolerated. No scalp relapses were seen among 30 patients (22% of all inclusions) having disease relapse.

**Conclusion:**

Adjuvant treatment with docetaxel (CD ≥ 400 mmg/m^2^) is associated with a significant rate of grade 2 PA, leading to wearing a wig, in around 10% of patients. This toxicity was completely prevented with scalp cooling. Clinical Trial Reference: NCT00515762.

**Electronic supplementary material:**

The online version of this article (10.1007/s10549-018-4855-2) contains supplementary material, which is available to authorized users.

## Introduction

Alopecia is a common toxicity of anticancer drugs and is considered by the patient as the second worst side-effect of chemotherapy, after emesis [1]. Cytotoxic agents cause alopecia by inhibiting the mitotic process in anagen hair roots. Hair loss usually starts 2 weeks following the initiation of chemotherapy, and has been traditionally considered to be reversible in all cases, after 3–4 months of cessation of therapy [2]. However, the observation of persistent alopecia (PA) long time after the end of adjuvant docetaxel chemotherapy in some of our BC patients, prompted us to study the prevalence of this phenomenon in BC patients and to explore the ability of scalp hypothermia to prevent this toxicity.

## Materials and methods

The main aims of the study were the following:

1. To establish the prevalence of PA resulting from adjuvant docetaxel for BC.
2. To test the efficacy of scalp hypothermia in preventing docetaxel-associated PA.

In the prevalence study, the population consisted of consecutive BC patients seen in the outpatient clinics of a Spanish institution (Hospital Clinico San Carlos, Madrid, HCSC) using Taxotere (Sanofi-Aventis, Paris). In order to confirm the prevalence of PA after docetaxel, the study was extended later on to other two institution (Instituto de Investigación Sanitaria Gregorio Marañón, Madrid, IISGM and Hospital Clínico de Valencia, HCUV) in which several docetaxel generics were used. Selection criteria were the following:

1. BC patients with UICC stage I–III.
2. Patients on follow-up 1.5–5 years after adjuvant/neoadjuvant anthracycline or taxane chemotherapy, alone or followed by endocrine adjuvant therapy and/or trastuzumab.
3. No evidence of disease recurrence.

Alopecia was graded according to the criteria used in the Common Terminology Criteria for Adverse Events CTCAE v3.0 for acute (reversible) alopecia. Grade 1 PA was defined as weakening of hair or partial alopecia and not leading to the use of a wig, after at least 18 months from the end of adjuvant chemotherapy (ACT). Grade 2 PA was defined as complete alopecia that requires a wig after at least 18 months from the end of ACT. In the first institution (HCSC), both grade 1 and 2 PA were recorded and a subset of 144 patients treated with adjuvant endocrine aromatase therapy (without chemotherapy) was also included to ascertain the contribution of this therapy to PA. In the two other institutions, only grade 2 PA was recorded.

Patient (age, menopausal status), tumor (stage, hormone receptors, her2/neu status), and treatment characteristics (chemotherapy schedules, radiation, endocrine treatment, trastuzumab) and time from surgery and from conclusion of adjuvant therapy were recorded. Patients were interviewed and explored to detect PA.

Patients with PA in one institution (HCSC) were examined by a dermatologist who analyzed the pattern of alopecia and performed a 4 mm punch biopsy of scalp for microscopic examination.

### Prevention study

The prevention study (NCT00515762) was carried out in one of the participating institutions (HCSC) in a series of consecutive patients receiving one of the two following adjuvant regimens:

1. AC/EC→T: doxorubicin (60 mg/m^2^) or epirubicin (90 mg/m^2^) in combination with cyclophosphamide (600 mg/m^2^) for 4 cycles followed by docetaxel (100 mg/m^2^ × 4 cycles).
2. TAC: docetaxel 75 mg/m^2^, doxorubicine 50 mg/m^2^, cyclophosphamide 500 mg/m^2^, IV, every 3 weeks × 6 cycles.

Taxotere® (Sanofi-Aventis, Paris) was the only docetaxel used in this trial. Patients receiving AC/EC→T started prophylactic hypothermia in the docetaxel part of chemotherapy. Patients receiving TAC chemotherapy had scalp hypothermia from the first cycle on.

A frozen hypothermic scalp cap (Elasto-gel) maintained at stable − 25 °C, was placed on the patient scalp 15 min before docetaxel administration, and replaced by a second one 45 min later. The ELASTO-GEL hypothermia cap is a medical device available in Europe (European Directive 93/42/EEC) and US, (Icewraps, Escondido, CA, USA) made of a seamless flexible material containing a glycerin-based hydrogel that remains flexible even at very low temperatures (− 30 °C). The cap supplies local cooling and its use is indicated to prevent chemotherapy-induced alopecia in patients with cancer.

The cap was maintained until 30 min after the end of docetaxel infusion. This procedure was repeated each time the docetaxel treatment was administered until the end of chemotherapy. Before the first infusion of chemotherapy, a baseline assessment of the degree of alopecia was conducted. Cap comfort and tolerability were assessed 1 h after completing each infusion of the treatment. Follow-up visits took place every 6 months after treatment has ended in order to determine the percentage of patients with persistent alopecia.

Sample calculation was based on the results of the initial prevalence study carried out at the HCSC, in which around 50% of patients receiving TAC or anthracyclines followed by docetaxel without any method of prevention had grade 1 or 2 PA. According to the previous bibliography [3], we hypothesized that scalp hypothermia will reduce the alopecia rate to 20%. To achieve a potency of 99% for detecting differences with the null hypothesis comparator (*Ho: p* = 0.50), using a bilateral Chi-squared test with a 5% level of significance (*alpha* = 0.05), 41 fully evaluable patients were required. Considering that evaluable patients should have received a cumulative docetaxel dose of ≥ 400 mg/m^2^, an estimated drop-out rate of 30% over the study course was expected (including patients stopping therapy or with significant dose reductions) and, therefore, 59 patients were estimated to be included to obtain 41 patients for full assessment.

In 2008, after the enrolment of 59 patients, it was evident that the statistical assumption of the trial (incidence of PA with scalp hypothermia of 20%) was erroneous, since no single patient presented this side effect with scalp hypothermia. No significant side effects with ELASTO-GEL had been recorded either. Therefore, the protocol was amended to increase the trial sample size to 120 evaluable patients, to have a better estimation of the efficacy and tolerance of the method. A final sample size of 150 patients (including dropouts) was then established.

The patients were follow-up every 6 months during the first 5 years and yearly afterwards in order to evaluate scalp hair recovery.

The studies were approved by the Ethical Committees of the participating institutions. Patients signed an informed consent before being enrolling. Pictures of alopecia were reproduced (enmasked) with permission of the patients.

## Results

### Prevalence study

#### Clinical findings

**Fig. 1:**
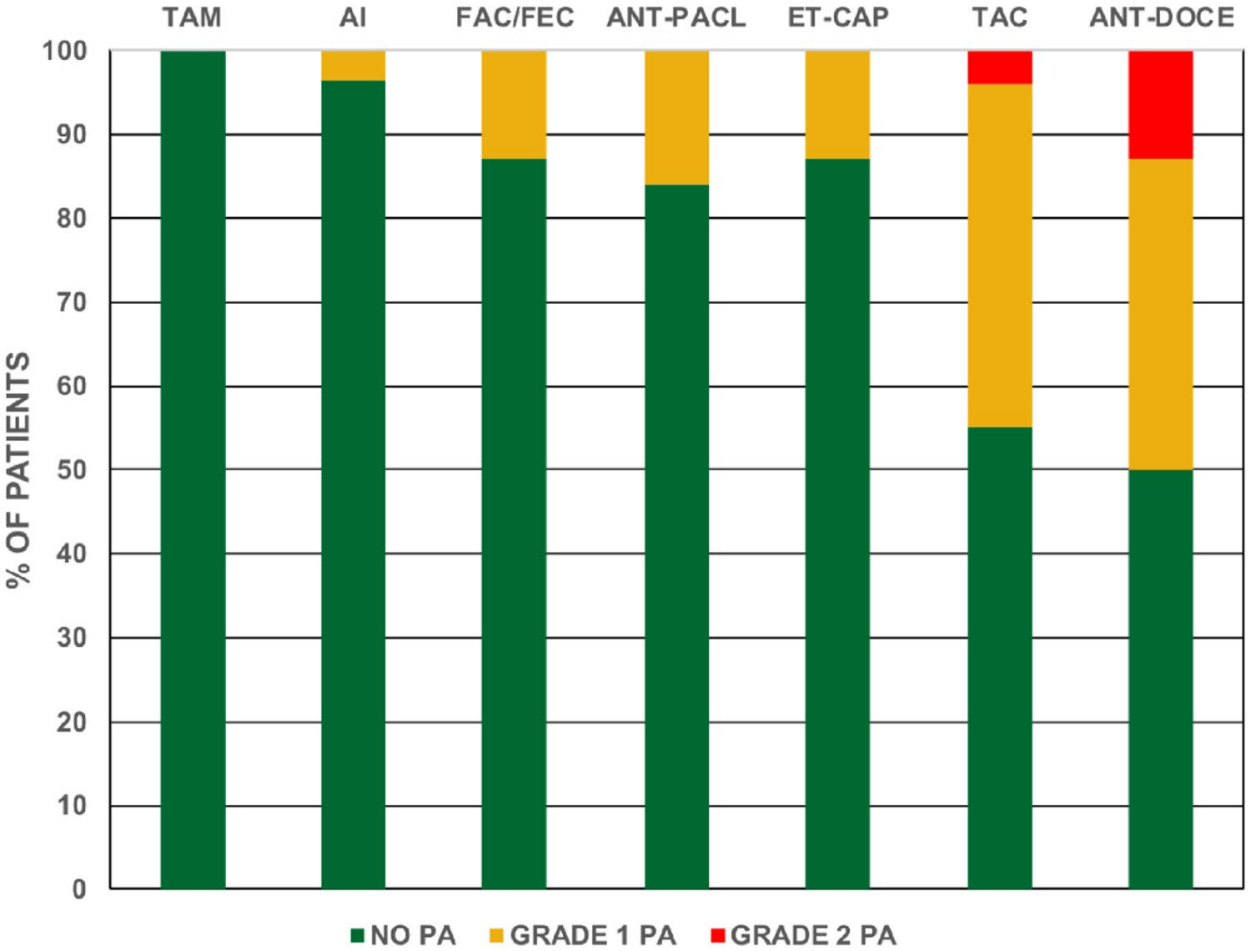
Persistent alopecia (PA) in patients treated with chemotherapy or endocrine therapy in one of the institutions (HCSC). TAM: tamoxifen; AI: aromatase inhibitors (no chemotherapy); FAC/FEC: 5-fluorouracil,doxorubicin or epirubicin, cyclophosphamide × 6 cycles; ANT-PACL: anthracyclines × 4 cycles followed by paclitaxel; ET-CAP: epirubicin plus docetaxel 75 mg/m2 × 4 cycles, followed by capecitabine; TAC: docetaxel 75 mg/m^2^, doxorubicin, cyclophosphamide × 6 cycles; ANT-DOCE: anthracyclines × 4 cycles followed by docetaxel 100 mg/m^2^ × 4 cycles; Around two-thirds of the patients treated with adjuvant chemotherapy received endocrine therapy with tamoxifen, aromatase inhibitors, or both afterwards

The prevalence study was initially performed at HCSC between December 2005 and May 2006. 492 patients (58% postmenopausal, 42% premenopausal, median age 53 years) were interviewed and explored (Table 1). Grade 1 PA was found with all chemotherapy regimens (variable proportions) and aromatase inhibitors (3.5% of cases), but not with tamoxifen (Fig. 1). Patients treated with docetaxel regimens (cumulative dose (CD) ≥ 400 mmg/m^2^) presented with a significantly higher prevalence of grade 1 PA (33–52%) and were the only ones having grade 2 PA (5–12%). The prevalence grade 2 PA with docetaxel was confirmed at two additional Spanish institutions. These institutions joined the prevalent study later, to confirm the prevalence of grade 2 PA (IISGM Dec 2009-August 2012 and HCV March 2014-August 2014). Minor forms of PA (grade 1) were not collected, since they were more difficult to agree upon in a homogenous way. None of the interviewed patients had scalp hypothermia during chemotherapy administration.

**Table 1:** Prevalence study

Number of patients	492
Median age (years, range)	53 (26–76)
Menopausal status	
Postmenopausal	58%
Premenopausal	42%
Adjuvant therapy	
FAC/FEC/AC/EC × 6 cycles	0/148
FAC/FEC/AC/EC × 4 cycles → weekly paclitaxel × 8–12 cycles	0/29
Epirubicin 90 mg/m^2^ plus docetaxel 75 mg/m^2^ × 4 cycles → Capecitabine × 4 cycles	0/31
TC × 4 cycles	0
TC × 6 cycles	0
TAC × 6 cycles	4/74 (5.4%)
TCH × 6 cycles	0
A/E+/−C × 4 cy → docetaxel 100 mg/m^2^ × 4 cycles +/−trastuzumab	8/66 (12%)
Tamoxifen^a^	0/57
Aromatase inhibitors^b^	0/87

Patients/treatment characteristics

*HCSC* Hospital Clinico San Carlos, Madrid; *FAC* 5-fluorouracil, doxorubicin, cyclophosphamide; *FEC*, 5-fluorouracil, doxorubicin, cyclophosphamide; AC/EC, doxorubicin or epirubicin plus cyclophosphamide; *TC* docetaxel 75 mg/m^2^ plus cyclophosphamide; TAC, docetaxel 75 mg/m^2^, doxorubicin, cyclophosphamide; *TCH*, docetaxel 75 mg/m^2^, carboplatin AUC 6, trastuzumab

^a^Without prior adjuvant chemotherapy

^b^Without prior adjuvant chemotherapy; some patients included in this group have received tamoxifen followed by an aromatase inhibitor

Taking together the three institutions, grade 2 PA was seen in patients receiving docetaxel regimens with a CD ≥ 400 mmg/m^2^ (36/358, 10.06%, 95% CI 7.36–13.61), but not in 59 patients with docetaxel regimens reaching lower CD of the drug (300 mg/m^2^), or chemotherapy regimens not containing docetaxel (*n* = *306*). Weekly paclitaxel was not associated with grade 2 PA. The incidence of docetaxel-induced grade 2 PA was similar in patients with (22/221, 9.96%) and without hormonal therapy (14/137, 10.2%). Trastuzumab did not increase the incidence of docetaxel-induced PA (Table 1b in the Supplement). The median follow-up of patients following the conclusion of chemotherapy was 43 months (range 18–60 months).

**Fig. 2:**
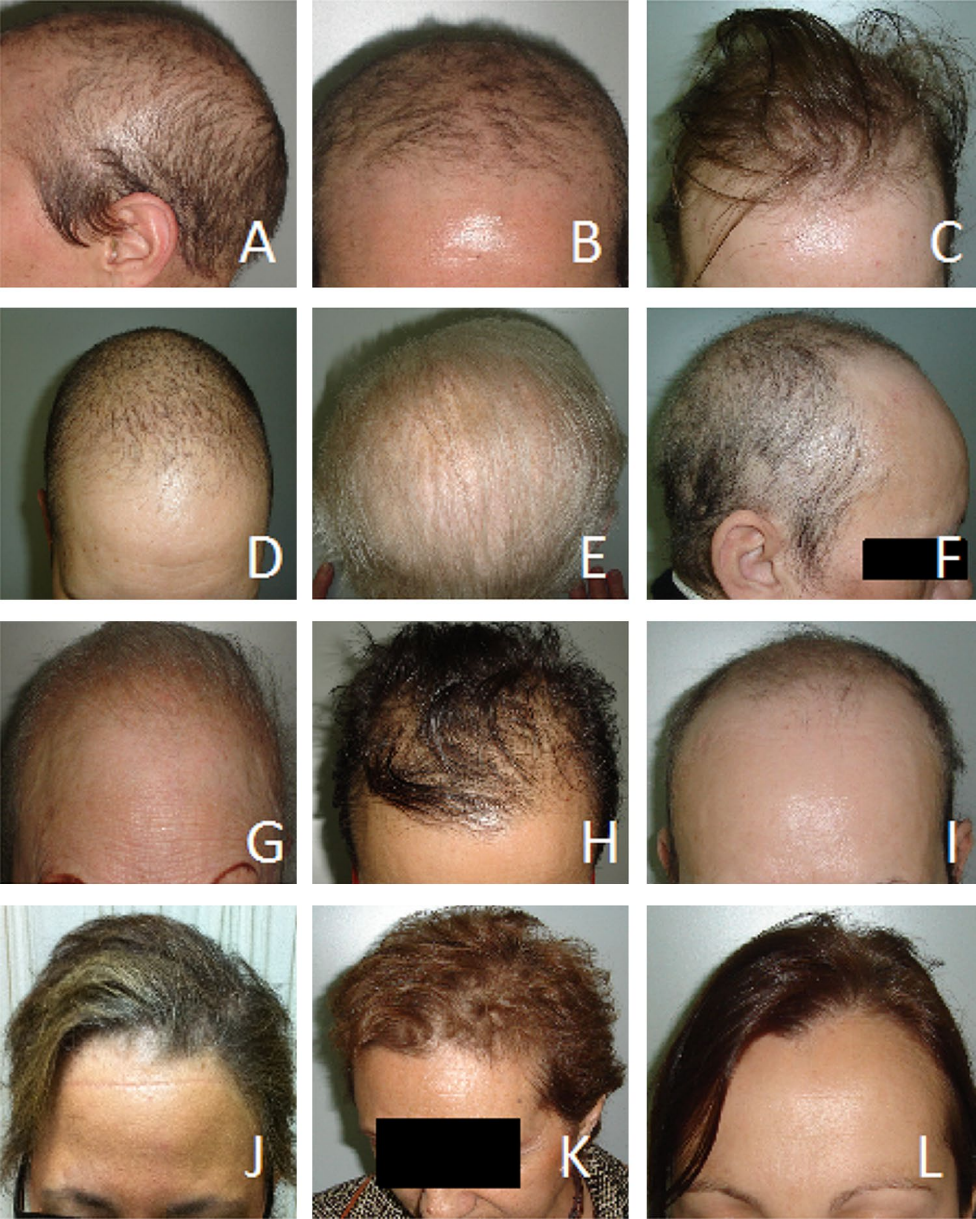
Persistent alopecina after 46 to 120 months following the conclusion of docetaxel chemotherapy. Panels **a**–**i**: Grade 2 Persistent Alopecia. Panels **j**–**l**: Grade 1 Persistent Alopecia (with permission from the patients)

Clinically, patients with grade 2 PA showed a practically complete loss of hair density (Fig. 2, panels a to i) and reported no significant improvement of hair growth over time, after a median follow-up of 48 months. Ten of these patients from HCSC have been followed up now for more than 10 years and did not show any sign of hair recovery in spite of the use of multiple dermatological therapies (including topical minoxidil at 5% in all cases).

### Pathology findings

Punch biopsy specimens were obtained from 10 patients with PMA treated with AC followed by docetaxel or with TAC at HCSC. All samples showed a marked reduction in large, terminal hairs with a reciprocal increase in small, vellus-like hairs (miniaturization of follicles); features very similar to those observed in androgenic alopecia. Neither significant peri-follicle inflammation nor fibrosis was noted (Fig. 3, in the Supplement).

### Prevention study: scalp hypothermia

Between March 2006 and September 2009, 136 operable BC patients scheduled to receive docetaxel at CD ≥ 400 mg/m^2^, either as a combination regimen (TAC) or as a single agent after four courses of doxorubicin/epirubicine plus cyclophosphamide, consented to enter the prevention study in HCSC. Patient characteristics are shown in Table 2.

**Table 2:** Intervention study (scalp hypothermia)

Characteristics and treatment	Number of patients (%)
Total number enrolled	136 (100%)
Menopausal status	
Premenopausal	57 (41.9%)
Postmenopausal	79 (58.1%)
Age (years)	
Median	53
Range	28–84
Chemotherapy régimen	
AC/EC → docetaxel	90 (66.2%)
TAC	46 (33.8%)
Actual docetaxel cumulative dose administered (mg/m^2^)	
Range	160–450
< 400	16 (11.8%)
400	81 (59.6%)
> 400	39 (28.7%)
Docetaxel dose modifications	
Reduction	10 (7.4%)
Interruption	6 (4.4%)
Adjuvant trastuzumab	
Yes	45 (38.8%)
No	71 (61.2%)
Adjuvant hormonal therapy	
Tamoxifen (+/−LHRH analogs)	37 (27%)
Aromatase inhibitors (+/−tamoxifen+/−LHRH analogs)	50 (36.8%)
None	49 (36%)

Patients characteristics and treatment

One hundred and twenty patients received docetaxel CD ≥ 400 mg/m^2^ and were considered evaluable for study purposes. The remaining patients did not reach the threshold docetaxel dose due to docetaxel interruption (6 patients) or dose reductions (10 patients).

After a median follow-up of 8 years, only a single patient among those evaluable presented with grade 1 PA (0.8%, 95% CI 0.15–4.56%). She was a 50 year old patient treated with AC followed by a CD of docetaxel of 400 mg/m^2^, followed by 5 years of letrozol. No cases of grade 2 PA were recorded. None of 16 non-evaluable patients (docetaxel dose < 400 mg/m^2^) presented any kind of PA.

BC relapses were seen in 30 patients (22% of all inclusions). Main sites of relapses were preserved breast (*n* = *1*), locoregional lymph nodes (*n* = *4*), bone (*n* = *16*), and visceral relapses (*n* = *9*). No scalp relapses were observed, neither isolated nor in association with other metastatic sites.

The tolerance to ELASTO-GEL device was good. Thirteen patients (10%) complained of mild headache, but all could finish the scheduled procedure.

## Discussion

Our study showed that PA is a significant problem for BC patients who have had received docetaxel-containing regimens; a significant percentage of patients is affected, and with no signs of recovery even after several years of follow-up. Alopecia is one of the most distressing side-effects of ACH and has been traditionally considered to be reversible at the conclusion of treatment BC [2]. Only a few cases of PA after chemotherapy had being described in the past, usually following high-dose chemotherapy with bone marrow or peripheral stem cell support [4, 5].

Several randomized phase III trials conducted during the decade of the 90s had shown than taxane-containing regimens are superior to classical anthracycline-containing chemotherapy [6, 7] and, therefore, these regimens have been considered the standard ACT for BC in most countries.

However, many of the taxane-containing regimens are also more toxic than the older regimens. The acute toxicity of docetaxel to skin and skin annexes have been described in previous clinical trials, and include erythema and skin desquamation of the limbs and nail changes in a considerable proportion of patients [8–10]. The skin toxicity incidence in phase III trials with 100 mg/m^2^ docetaxel doses ranged from 32 to 37%, with 4 to 4.5% of patients having grade 3–4 toxicity. Additionally, nearly 40% of patients had nail disorders.

Significant alopecia had been reported in 74.2% of docetaxel-treated patients [10], but had been described as systematically reversible. In 2001, Nabholtz et al. described, for the first time, four patients with long-lasting (> 2 years) PA following TAC (docetaxel, doxorubicin, cyclophosphamide) chemotherapy for metastatic BC [11], the finding having generated little attention at that time. In 2006, Sedlacek reported, the phenomenon of PA following ACT for BC [12]. The study retrospectively reviewed hair recovery in 496 consecutive patients who, over a period of 11 years, had been treated with ACT. All patients had at least one year of follow-up after therapy. The average time from the last chemotherapy dose was 48 months (range 19–85 months). The study defined PA as hair re-growth of < 50% of the pre-chemotherapy amount of hair, as judged by both the patient and the investigator (similar to grade 2 alopecia according to CTCAE v4.0). The chemotherapy regimens in this study were classified in 3 categories: group A (doxorubicin regimens without taxanes); group B (regimens including doxorubicin + paclitaxel); and group C (regimens including doxorubicin + docetaxel: AC followed by docetaxel, doxorubicin + docetaxel, TAC, AC followed by docetaxel + capecitabine, docetaxel + doxorubicin followed by CAF, CAF followed by docetaxel). No PA was found in groups 1 and 2, whereas it was present in 6.3% (7/112) of patients in group C. All patients with persistent significant alopecia had received AC followed by docetaxel (100 mg/m^2^).

Our study showed that grade 1 PA is present in a small proportion of patients following most regimens of ACT and/or aromatase inhibitors, the majority of them in postmenopausal patients. Progressive spontaneous alopecia is not an infrequent finding in postmenopausal women. Medications, concomitant chronic diseases, telogen effluvium, and, mostly, female pattern hair loss (FMHL) are frequent causes of alopecia in women post-menarche [13]. Aromatase inhibitors have also been reported as a cause of male pattern hair loss [14] and this was also seen in our study. Although causes other than chemotherapy itself can be involved in PA, the high proportion of patients having grade 1 PA (35–50%) after regimens including docetaxel at CD ≥ 400 mg/m^2^ suggests an ethiologic role for this drug. Besides, we found grade 2 PA only in patients treated with docetaxel regimens achieving the same cumulative dose of the drug being recorded in 10.06% of this population (95% CI 7.36–13.61%).

The pathogenesis of PMA following docetaxel is not known. Our study suggests that docetaxel can cause permanent damage of hair follicles. Punch biopsy specimens obtained from 10 patients with PMA showed an unspecific pattern of androgenic-like alopecia, with marked reduction in large, terminal hairs.

Kluger et al. [15] reported 21 Caucasian BC patients who presented PA after docetaxel adjuvant therapy, showing a moderate or intense androgenetic-like pattern of scalp alopecia. PA was defined as absent or incomplete hair regrowth at ≥ 6 months post chemotherapy. This description probably correspond to cases of grade 1 or 2 PA. Biopsy specimen examinations were normal or displayed the androgenetic-like pattern, as in our study. The chemotherapy protocols included docetaxel at CD of 300 mg/m^2^ (12 patients) or ≥ 400 mg/m^2^ (8 cases). Fonia et al. [16] described 10 patients with persistent alopecia following different combination regimens including docetaxel. The histopathologic study showed features of non-scarring alopecia with hair follicle units preservation, reduced hair density, and increased vellus hair follicles, a pattern very similar to that seen in our patients.

In recent years, renewed interest in chemotherapy-induced hair loss prevention has arised. Two recent trials have supported the ability of scalp hypothermia to reduce or completely prevent chemotherapy-induced alopecia [17, 18]. However, these trials were aimed at preventing chemotherapy-induced acute (reversible) alopecia. The aim of our preventive study was different, the prevention of PA following docetaxel. The rationale of our study was based on the success of chilled gloves in reducing nail and cutaneous toxicity of the hand associated with docetaxel therapy [19].

Our study showed that scalp cooling is very effective in preventing persistent alopecia following docetaxel. No patient experienced grade 2 PA and only one patient (0.8%, 95% CI 0.15–4.56%) had grade 1 PA.

A concern that has reduced in the past the use of scalp hypothermia for preservation of hair during chemotherapy is the risk of scalp metastases. The reduced exposition of the scalp to docetaxel or other chemotherapy drugs could facilitate the persistence of tumor cells, leading to scalp metastases months to years later [20]. However, a recent meta-analysis has found that the incidence of scalp metastases is very low, regardless of scalp cooling: 0.61% (95% CI 0.32–1.1%) with scalp cooling versus 0.41% (95% CI 0.13–0.94%) in a control group (*p* = 0.43) [21]. Therefore, scalp cooling is unlikely to be a cause of increase incidence of scalp metastases.

In conclusion, PA following adjuvant docetaxel chemotherapy is a significant toxicity of the drug, particularly when administered at CD of ≥ 400 mg/m^2^. Scalp cooling is very effective in preventing this undesirable side effect of docetaxel.

## Electronic supplementary material

Below is the link to the electronic supplementary material.

Supplementary material 1 (DOCX 1075 KB)
