# Supplementary material for: Persistent major alopecia following adjuvant docetaxel for breast cancer: incidence, characteristics, and prevention with scalp cooling
Source: Breast Cancer Res Treat. 2018 Jun 19;171(3):627–34. doi: 10.1007/s10549-018-4855-2 (PMC6133184; doi:10.1007/s10549-018-4855-2)
Supplement: Supplementary file 1 — Supplementary material 1 (DOCX 1075 KB) [file 10549_2018_4855_MOESM1_ESM.docx]

# APPENDICES/SUPPLEMENTAL MATERIAL

ON LINE ONLY: FIGURE 3

Microscopic image of scalp biopsy from a patient with PA grade 2. A marked

reduction in large, terminal hairs with a reciprocal increase in small, vellus-like hairs

(miniaturization of follicles*) is evident. Hematoxylin and eosin staining, original

magnification x 10

ON LINE ONLY: **SUPPLEMENTARY TABLE 1B**

Prevalence study. Patients/treatement characteristics and prevalence of persistent major alopecia (PMA).

|  | INSTITUTION | | | |
| --- | --- | --- | --- | --- |
|  | HCSC^1^ | IISGM^2^ | HCUV^3^ | All |
| Number of patients | 492 | 228 | 147 | 867 |
| Median Age (years, range) | 53 (26-76) | 53 (26-79) | 52 (35-84) | 53 (26-84) |
| postmenopausal  premenopausal | 58%  42% | 55.5%  44.5% | 60.3%  39.7% | 58%  42% |
| MAIN ADJUVANT THERAPY | PATIENTS WITH PMA | | | |
| FAC/FEC/AC/EC x 6 cycles | 0/148 | 0/36 | 0/42 | 0/226 |
| FAC/FEC/AC/EC x 4 cycles🡪weekly paclitaxel x 8-12 cycles | 0/29 | 0/34 | 0/17 | 0/80 |
| Epirubicin 90 mg/m2 plus docetaxel 75 mg/m2 x 4 cycles🡪Capecitabine x 4 cycles | 0/31 | 0 | 0 | 0/31 |
| TC x 4 cycles | 0 | 0/19 | 0/9 | 0/28 |
| TC x 6 cycles | 0 | 2/30  (7%) | 3/18  (17%) | 5/48  (10.4%) |
| TAC x 6 cycles | 4/74  (5.4%) | 3/32  (9.4%) | 5/43  (12%) | 12/149  (8%) |
| TCH x 6 cycles | 0 | 2/25  (8%) | 1/6  (12%) | 3/31  (9.7%) |
| A/E+/-C x 4 cy🡪docetaxel 100mg/m2 x 4 cycles +/-trastuzumab | 8/66  (12%) | 7/52  (13.5%) | 1/12  (8.3%) | 16/130 (12.3%) |
| tamoxifen^*^ | 0/57 | 0 | 0 | 0/57 |
| aromatase inhibitors^**^ | 0/87 | 0 | 0 | 0/87 |

**Prevalence study. Patients/treatement characteristics and prevalence of persistent major alopecia (PMA).** HCSC: Hospital Clinico San Carlos, Madrid; IISGM: Instituto de Investigación Sanitaria Gregorio Marañón; HCUV: Hospital Clínico de Valencia, Spain); FAC: 5-fluorouracil, doxorubicin, cyclophosphamide; FEC: 5-fluorouracil, doxorubicin, cyclophosphamide; AC/EC:doxorubicin or epirubicin plus cyclophosphamide; TC: docetaxel 75 mg/m2 plus cyclophsphamide; TAC: docetaxel 75 mg/m2, doxorubicin, cyclophosphamide; TCH: docetaxel 75 mg/m2, carboplatin AUC 6, trastuzumab;
